# Determining the Number of Attributes in Cognitive Diagnosis Modeling

**DOI:** 10.3389/fpsyg.2021.614470

**Published:** 2021-02-15

**Authors:** Pablo Nájera, Francisco José Abad, Miguel A. Sorrel

**Affiliations:** Department of Social Psychology and Methodology, Faculty of Psychology, Autonomous University of Madrid, Madrid, Spain

**Keywords:** cognitive diagnostic models, dimensionality assessment, parallel analysis, machine learning, model comparison, Q-matrix validation

## Abstract

Cognitive diagnosis models (CDMs) allow classifying respondents into a set of discrete attribute profiles. The internal structure of the test is determined in a Q-matrix, whose correct specification is necessary to achieve an accurate attribute profile classification. Several empirical Q-matrix estimation and validation methods have been proposed with the aim of providing well-specified Q-matrices. However, these methods require the number of attributes to be set in advance. No systematic studies about CDMs dimensionality assessment have been conducted, which contrasts with the vast existing literature for the factor analysis framework. To address this gap, the present study evaluates the performance of several dimensionality assessment methods from the factor analysis literature in determining the number of attributes in the context of CDMs. The explored methods were parallel analysis, minimum average partial, very simple structure, DETECT, empirical Kaiser criterion, exploratory graph analysis, and a machine learning factor forest model. Additionally, a model comparison approach was considered, which consists in comparing the model-fit of empirically estimated Q-matrices. The performance of these methods was assessed by means of a comprehensive simulation study that included different generating number of attributes, item qualities, sample sizes, ratios of the number of items to attribute, correlations among the attributes, attributes thresholds, and generating CDM. Results showed that parallel analysis (with Pearson correlations and mean eigenvalue criterion), factor forest model, and model comparison (with AIC) are suitable alternatives to determine the number of attributes in CDM applications, with an overall percentage of correct estimates above 76% of the conditions. The accuracy increased to 97% when these three methods agreed on the number of attributes. In short, the present study supports the use of three methods in assessing the dimensionality of CDMs. This will allow to test the assumption of correct dimensionality present in the Q-matrix estimation and validation methods, as well as to gather evidence of validity to support the use of the scores obtained with these models. The findings of this study are illustrated using real data from an intelligence test to provide guidelines for assessing the dimensionality of CDM data in applied settings.

## Introduction

The correct specification of the internal structure is arguably the key issue in the formulation process of a measurement model. Hence, it is not surprising that the determination of the number of factors has been regarded as the most crucial decision in the context of exploratory factor analysis (EFA; e.g., Garrido et al., 2013; Preacher et al., 2013). Since the very first proposals to address this issue, such as the eigenvalue-higher-than-one rule or Kaiser-Guttman criterion (Guttman, 1954; Kaiser, 1960), many methods have been developed for assessing the dimensionality in EFA. Despite the longevity of this subject of study, the fact that it is still a current research topic (e.g., Auerswald and Moshagen, 2019; Finch, 2020) is a sign of both its relevance and complexity.

In contrast to the vast research in the EFA framework, dimensionality assessment remains unexplored for other measurement models. This is the case of cognitive diagnosis models (CDMs). CDMs are restricted latent class models in which the latent variables or *attributes* are discrete, usually dichotomous. The popularity of CDMs has increased in the last years, especially in the educational field, because of their ability to provide a fine-grained information about the examinees' mastery or non-mastery of certain skills, cognitive processes, or competences (de la Torre and Minchen, 2014). However, CDM applications are not restricted to educational settings, and they have been employed for the study of psychological disorders (Templin and Henson, 2006; de la Torre et al., 2018) or staff selection processes (García et al., 2014; Sorrel et al., 2016).

A required input for CDMs is the *Q-matrix* (Tatsuoka, 1983). It has dimensions *J* items × *K* attributes, in which each *q-entry* (*q*_*jk*_) can adopt a value of 1 or 0, depending on whether attribute *k* is relevant to measure item *j* or not, respectively. Hence, the Q-matrix determines the internal structure of the test, and its correct specification is fundamental to obtain accurate structural parameter estimates and, subsequently, an accurate classification of examinees' latent classes or *attribute profiles* (Rupp and Templin, 2008; Gao et al., 2017). However, the Q-matrix construction process is usually conducted by domain experts (e.g., Sorrel et al., 2016). This process is subjective in nature and susceptible to specification errors (Rupp and Templin, 2008; de la Torre and Chiu, 2016). To address this, several Q-matrix estimation and validation methods have been proposed in the recent years with the aim of providing empirical support to its specification. On the one hand, empirical Q-matrix estimation methods rely solely on the data to specify the Q-matrix. For instance, Xu and Shang (2018) developed a likelihood-based estimation method, which aims to find the Q-matrix that shows the best fit while controlling for model complexity. Additionally, Wang et al. (2018) proposed the *discrete factor loading* (DFL) method, which consists in conducting an EFA and dichotomizing the factor loading matrix up to some criterion (e.g., row or column means). On the other hand, empirical Q-matrix validation methods aim to correct a provisional, potentially misspecified Q-matrix based on both its original specification and the data. For instance, the *stepwise* method (Ma and de la Torre, 2020a) is based on the Wald test to select the q-entries that are statistically necessary for each item, while the *general discrimination index* method (de la Torre and Chiu, 2016) and the *Hull* method (Nájera et al., 2020) aim to find, for each item, the simplest *q-vector* specification that leads to an adequate discrimination between latent classes. These methods serve as a useful tool for applied researchers, who can obtain empirical evidence of the validity of their Q-matrices (e.g., Sorrel et al., 2016).

Despite their usefulness, the Q-matrix estimation and validation methods share an important common drawback, which is assuming that the number of attributes specified by the researcher is correct (Xu and Shang, 2018; Nájera et al., 2020). Few studies have tentatively conducted either a parallel analysis (Robitzsch and George, 2019) or model-fit comparison (Xu and Shang, 2018) to explore the dimensionality of the Q-matrix. However, to the authors' knowledge, there is a lack of systematic studies on the empirical estimation of the number of attributes in CDMs. The main objective of the present research is precisely to compare the performance of a comprehensive set of dimensionality assessment methods in determining the number of attributes. The remaining of the paper is laid out as follows. First, a description of two popular CDMs is provided. Second, a wide selection of EFA dimensionality assessment methods is described. Third, an additional method for assessing the number of attributes in CDMs is presented. Fourth, the design and results from an exhaustive simulation study are provided. Fifth, real CDM data are used for illustrating the functioning of the dimensionality assessment methods. Finally, practical implications and future research lines are discussed.

## The Dina and G-Dina Models

CDMs can be broadly separated into general and reduced, specific models. General CDMs are saturated models that subsume most of the reduced CDMs. They include more parameters and, consequently, provide a better model-data fit in absolute terms. As a counterpoint, their estimation is more challenging. Thus, reduced CDMs are often a handy alternative to applied settings because of their simplicity, which favors both their estimation and interpretation. Let denote by $K_{j}^{*}$ the number of required attributes for item *j*. Under the *deterministic inputs, noisy “and” gate* model (DINA; Junker and Sijtsma, 2001), which is a conjunctive reduced CDM, there are only two parameters per item regardless of $K_{j}^{*}$: the *guessing* parameter (*g*_*j*_), which is the probability of correctly answering item *j* for those examinees that do not master, at least, one of the required attributes, and the *slip* parameter (*s*_*j*_), which is the probability of failing item *j* for those examinees that master all the attributes involved. The probability of correctly answering item *j* given latent class *l* is given by

(1) $$\begin{array}{l} P_{j}\left(\alpha_{l}\right)=g_{j}^{1-\eta_{lj}}{\left(1-s_{j}\right)}^{\eta_{lj}} \end{array}$$

where η_*lj*_ equals 1 if examinees in latent class *l* master all the attributes required by item *j*, and 0 otherwise.

**Figure 1:**
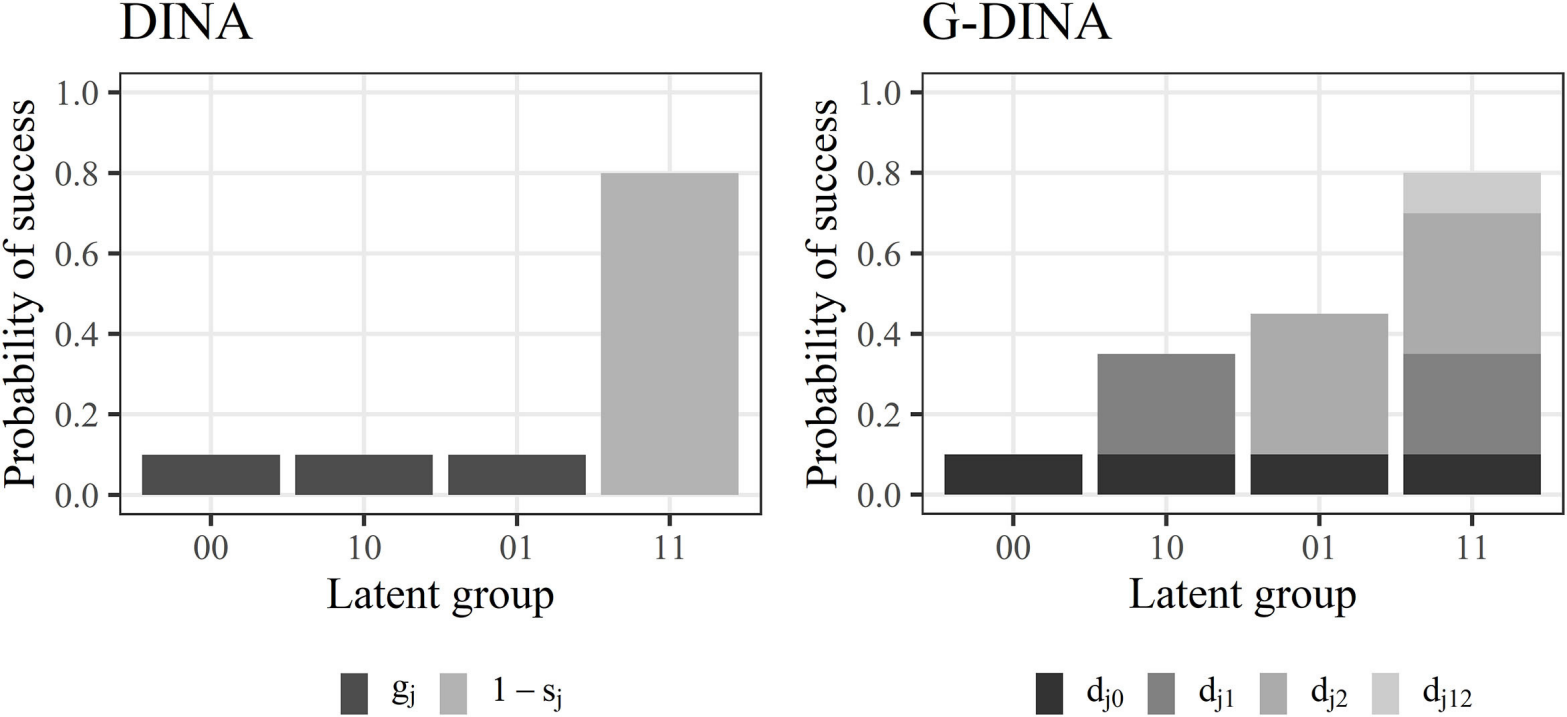
Illustration of item parameters and probabilities of success of the different latent groups for the DINA and G-DINA models involving 2 attributes (*K* = 2).

The *generalized* DINA model (G-DINA; de la Torre, 2011) is a general CDM, in which the probability of correctly answering item *j* for latent class *l* is given by the sum of the main effects of the required attributes and their interaction effects (in addition to the intercept):

(2) $$\begin{array}{l} P_{j}\left(\alpha_{l}^{*}\right)=\delta_{j0}+\overset{K_{j}^{*}}{\underset{k=1}{\sum }}\delta_{jk}\alpha_{lk}+\overset{K_{j}^{*}}{\underset{k^{\prime }=k+1}{\sum }}\overset{K_{j}^{*}-1}{\underset{k=1}{\sum }}\delta_{jkk^{\prime }}\alpha_{lkk^{\prime }}\ldots \\ +\delta_{j12\ldots K_{j}^{*}}\overset{K_{j}^{*}}{\underset{k=1}{\prod }}\alpha_{lk} \end{array}$$

where $\alpha_{l}^{*}$ is the reduced attribute profile whose elements are the ${K_{j}}^{*}$ required attributes for item *j*, δ_*j*0_ is the intercept for item *j*, δ_*jk*_ is the main effect due to α_*k*_, $\delta_{jkk^{\prime }}$ is the interaction effect due to α_*k*_ and $\alpha_{k^{\prime }}$, and $\delta_{j12\ldots K_{j}^{*}}$ is the interaction effect due to $\alpha_{1},\ldots ,\alpha_{K_{j}^{*}}$. Figure 1 depicts the probabilities of success of the four possible latent groups for an item requiring two attributes ($K_{j}^{*}$ = 2) under the DINA and G-DINA models. For the DINA model, the probability of success for the latent group that masters all attribute is high (*P*(11) = 1 − *s*_*j*_ = 1 − 0.2 = 0.8), while the probability of success for the remaining latent groups is very low (*P*(00) = *P*(10) = *P*(01) = *g*_*j*_ = 0.1). For the G-DINA model, the baseline probability (i.e., intercept) is also very low (*P*(00) = δ_*j*0_ = 0.1). The increment in the probability of success as a result of mastering the first attribute (*P*(10) = δ_*j*0_ + δ_*j*1_ = 0.1 + 0.25 = 0.35) is slightly lower than the one due to mastering the second attribute (*P*(01) = δ_*j*0_ + δ_*j*2_ = 0.1 + 0.35 = 0.45). Finally, although the interaction effect for both attributes is low (δ_*j*12_ = 0.1), the probability of success for the latent group that masters both attributes is high because the main effects are also considered (*P*(11) = δ_*j*0_ + δ_*j*1_ + δ_*j*2_ + δ_*j*12_ = 0.1 + 0.25 + 0.35 + 0.1 = 0.80).

## Dimensionality Assessment Methods

In the following, we provide a brief explanation of seven dimensionality assessment methods that were originally developed for determining the number of factors in EFA and will be explored in the present study.

### Parallel Analysis

Parallel analysis (PA; Horn, 1965) compares the eigenvalues extracted from the sample correlation matrix (i.e., sample eigenvalues) with the eigenvalues obtained from several randomly generated correlation matrices (i.e., reference eigenvalues). The number of sample eigenvalues that are higher than the average of their corresponding reference eigenvalues is retained as the number of factors. The 95th percentile has also been recommended rather than the mean to prevent from over-factoring (i.e., overestimate the number of factors). However, no differences have been found in recent simulation studies between both cutoff criteria (Crawford et al., 2010; Auerswald and Moshagen, 2019; Lim and Jahng, 2019). Additionally, polychoric correlations have been recommended when working with categorical variables. Although no differences have been found for non-skewed categorical data, polychoric correlations perform better with skewed data (Garrido et al., 2013) as long as the reference eigenvalues are computed considering the univariate category probabilities of the sample variables by, for instance, using random column permutation for generating the random samples (Lubbe, 2019). Finally, different extraction methods have been used to compute the eigenvalues: principal components analysis (Horn, 1965), principal axis factor analysis (Humphreys and Ilgen, 1969), or minimum rank factor analysis (Timmerman and Lorenzo-Seva, 2011). The original proposal by Horn has consistently shown the best performance across a wide range of conditions (Garrido et al., 2013; Auerswald and Moshagen, 2019; Lim and Jahng, 2019). Simulation studies have shown the superiority of PA above other dimensionality assessment methods. Thus, it is usually recommended and considered the gold standard (Garrido et al., 2013; Auerswald and Moshagen, 2019; Lim and Jahng, 2019; Finch, 2020). As a flaw, PA tends to under-factor (i.e., underestimate the number of factors) in conditions with low factor loadings or highly correlated factors (Garrido et al., 2013; Lim and Jahng, 2019).

### Minimum Average Partial

The minimum average partial (MAP; Velicer, 1976) method has also been recommended for determining the number of factors with continuous data (Peres-Neto et al., 2005). It is based on principal components analysis and the partial correlation matrix. The MAP method extracts one component at a time and computes the average of the squared partial correlations (MAP index). The MAP method relies on the rationale that extracting the first components, which explain most of the common variance, will result in a decrease of the MAP index. Once the relevant components have been partialled out, extracting the remaining ones (which are formed mainly by unique variance) will make the MAP index to increase again. The optimal number of components corresponds to the lowest MAP index. A variant where the MAP index is computed by averaging the fourth power of the partial correlation was proposed by Velicer et al. (2000). However, Garrido et al. (2011) recommended the use of the original squared partial correlations, in addition to polychoric correlations when categorical variables are involved. They found that MAP method performed poorly under certain unfavorable situations, such as low-quality items or small number of variables per factor, where the method showed a tendency to under-factor.

### Very Simple Structure

The very simple structure (VSS; Revelle and Rocklin, 1979) method was developed with the purpose of providing the best interpretable factor solution, understood as the absence of cross-loadings. In this procedure, a loading matrix with *K* factors is first estimated and rotated. Then, a simplified factor loading matrix (${{\text{S}}^{\prime }}_{vk}$) is obtained, given a prespecified complexity *v*. Namely, the *v* highest loadings for each item are retained and the remaining loadings are fixed to zero. Then, the residual correlation matrix is found by

(3) $${\bar{\text{R}}}_{vk}=\text{R}-{\text{S}}_{vk}\Phi_{\text{k}}\text{S}\prime_{vk}$$

where **R** is the observed correlation matrix and **Φ**_**k**_ is the factor correlation matrix. Then, the VSS index is computed as

(4) $$\begin{array}{l} VSS_{vk}=1-\frac{MS_{{\bar{\text{R}}}_{vk}}}{MS_{\text{R}}} \end{array}$$

where $MS_{{\bar{\text{R}}}_{vk}}$ and *MS*_**R**_ are the average of the squared residual and observed correlations, respectively. The VSS index is computed for each factor solution, and the highest VSS corresponds to the number of factors to retain. The main drawback of the procedure is that the researcher must prespecify a common expected complexity for all the items, which is usually *v* = 1 (VSS_1_; Revelle and Rocklin, 1979). In a recent simulation study, the VSS method obtained a poor performance under most conditions, over-factoring with uncorrelated factors and under-factoring with highly correlated factors (Golino and Epskamp, 2017).

### Dimensionality Evaluation to Enumerate Contributing Traits

The dimensionality evaluation to enumerate contributing traits (DETECT; Kim, 1994; Zhang and Stout, 1999; Zhang, 2007) method is a nonparametric procedure that follows two strong assumptions: first, a single “dominant” dimension underlies the item responses, and second, the residual common variance between the items follows a simple structure (i.e., without cross-loadings). The method estimates the covariances of item pairs conditioned to the raw item scores, which are used as a non-parametric approximation to the dominant dimension. If the data are essentially unidimensional, these conditional covariances will be close to zero. Otherwise, items measuring the same secondary dimension will have positive conditional covariances, and items measuring different secondary dimensions will have negative conditional covariances. The DETECT index is computed as

(5) $$\begin{array}{l} D\left(P\right)=\frac{1}{J\left(J-1\right)/2}\underset{j<j^{\prime }}{\sum }{\left(-1\right)}^{c_{jj^{\prime }}}\left({Ĉ}_{jj^{\prime }}-\bar{C}\right) \end{array}$$

where *P* represents a specific partitioning of items into clusters, ${Ĉ}_{jj^{\prime }}$ is the estimated conditional covariance between items *j* and *j*′, $\bar{C}$ is the average of the estimated conditional covariances, and $c_{jj^{\prime }}$ = 0 or 1 if items *j* and *j*′ are part of the same cluster or not, respectively. The method explores different number of dimensions and the one that obtains the highest DETECT index is retained. Furthermore, the method also provides which items measure which dimension. In a recent study, Bonifay et al. (2015) found that the DETECT method had a great performance at the population level, retaining the correct number of dimensions in 97% of the generated datasets with *N* = 10,000. As a limitation of the study, the authors only tested a scenario in which the generating model had no cross-loadings (i.e., simple structure).

### Empirical Kaiser Criterion

The empirical Kaiser criterion (EKC; Braeken and van Assen, 2017) is similar to PA in that the sample eigenvalues are compared to reference eigenvalues to determine the number of factors to retain. Here, reference eigenvalues are derived from the theoretical sampling distribution of eigenvalues, which is a Marčenko-Pastur distribution (Marčenko and Pastur, 1967) under the null hypothesis (i.e., non-correlated variables). The first reference eigenvalue depends only on the ratio of test length to sample size, while the subsequent reference eigenvalues consider the variance explained by the previous ones. The reference eigenvalues are coerced to be at least equal to 1, and thus it cannot suggest more factors than the Kaiser-Guttman criterion would. In fact, EKC is equivalent to the Kaiser-Guttman criterion at the population level. EKC has been found to perform similarly to PA with non-correlated variables, unidimensional models, and orthogonal factors models, while it outperformed PA with oblique factors models and short test lengths (Braeken and van Assen, 2017). The performance of EKC with non-continuous data remains unexplored.

### Exploratory Graph Analysis

EGA (Golino and Epskamp, 2017) is a recently developed technique that has emerged as a potential alternative for PA. EGA was first developed based on the Gaussian graphical model (GGM; Lauritzen, 1996), which is a network psychometric model in which the joint distribution of the variables is estimated by modeling the inverse of the variance-covariance matrix. The GGM is estimated using the least absolute shrinkage and selector operator (LASSO; Tibshirani, 1996), which is a penalization technique to avoid overfitting. Apart from EGA with GGM, Golino et al. (2020) recently proposed an EGA based on the triangulated maximally filtered graph approach (TMFG; Massara et al., 2016), which is not restricted to multivariate normal data. Regardless of the model (GGM or TMFG), in EGA each item is represented by a node and each edge connecting two nodes represents the association between the two items. Partial correlations are used for EGA with GGM, while any association measure can be used for EGA with TMFG. A strong edge between two nodes is interpreted as both items being caused by the same latent variable. A *walktrap* algorithm is then used to identify the number of clusters emerging from the edges, which will be the number of factors to retain. Furthermore, EGA also provides information about what items are included in what clusters, and clusters can be related to each other if their nodes are correlated. EGA with GGM seems to have an overall better performance than EGA with TMFG (Golino et al., 2020). EGA with GGM has been found to perform similarly to PA in most situations, with slightly worse results with low factor correlations, but better performance with highly correlated factors (Golino and Epskamp, 2017). On the other hand, EGA with TMFG tends to under-factor when there are many variables per factor or highly correlated factors (Golino et al., 2020).

### Factor Forest

Factor forest (FF; Goretzko and Bühner, 2020) is an extreme gradient boosting machine learning model that was trained to predict the optimal number of factors in EFA. Specifically, the model estimates the probability associated to different factor solutions and subsequently suggests the number of factors with the highest probability. As opposed to the previously described dimensionality assessment methods, the FF is not based on any particular theoretical psychometric background, and its purpose is to make accurate predictions based on a combination of empirical results obtained from the training datasets. This is commonly referred to as the “black box” character of the machine learning models (Goretzko and Bühner, 2020). In the original paper, the authors trained the FF model using a set of 181 features (e.g., eigenvalues, sample size, number of variables, Gini-coefficient, Kolm measure of inequality) while varying the sample size, primary and secondary loadings, number of factors, variables per factor, and factor correlations, through almost 500,000 datasets. The data were generated assuming multivariate normality. The FF model obtained very promising results, correctly estimating the number of factors in 99.30% of the evaluation datasets. The Kolm measure of inequality and the Gini-coefficient were the most influential features on the predictions of the model. It is remarkable that some evaluation conditions were different from those used in the training stage. Thus, the FF model trained in Goretzko and Bühner (2020) for the EFA framework will be explored in the present paper.

## Model-Fit Indices for Determining the Number of Attributes in CDM

All the aforementioned methods were developed with the purpose of assessing the number of factors in the EFA framework and, thus, their assumptions might not fit the nature of CDM data. Table 1 shows that some of the most important assumptions required by some of the methods might be usually violated when analyzing CDM data. There is, however, one additional procedure that can be applied to CDMs without any further assumptions: the model comparison approach based on model-fit indices. This approach has also been widely explored in EFA. Previous studies have shown that, even though the traditional cutoff points for some commonly used fit indices (e.g., CFI, RMSEA, SRMR) are not recommended for determining the number of factors (Garrido et al., 2016), the relative difference in fit indices between competing models might even outperform PA under some conditions, such as small loadings, categorical data (Finch, 2020), or orthogonal factors (Lorenzo-Seva et al., 2011). Additionally, Preacher et al. (2013) recommended to use AIC for extracting the number of factors whenever the goal of the research was to find a model with an optimal parsimony-fit balance, while they recommended RMSEA whenever the goal was to retain the true, generating number of factors.

**Table 1:** Dimensionality assessment methods assumptions.

**Method**	**Based on**	**Latent variable**	**Essential unidim**.	**Simple structure**
PA	Eigenvalues	No	**No**	**No**
MAP	Partial correlations	No	**No**	**No**
VSS^a^	Factor loadings	Continuous	**No**	Yes
DETECT	Conditional covariances	Continuous	Yes	Yes
EKC	Eigenvalues	No	**No**	**No**
EGA	Network psychometrics	No	**No**	**No**
MC	Model-fit indices	**Discrete**	**No**	**No**

*The methods assumptions that are aligned with cognitive diagnosis modeling characteristics are highlighted in bold. The factor forest (FF) model has not been included due to its dependence on the conditions employed for training the model. Essential unidim., essential unidimensionality; PA, parallel analysis with principal components extraction; MAP, minimum average partial; VSS, very simple structure; DETECT, dimensionality evaluation to enumerate contributing traits; EKC, empirical Kaiser criterion; EGA, exploratory graph analysis; MC, model comparison based on fit indices*.

a *Simple structure (understood as a single factor being measured by each item) is technically assumed only by VSS with complexity v = 1*.

In the CDM framework, relative and absolute fit indices have been used to select the most appropriate Q-matrix specification. Regarding relative model-fit indices, Kunina-Habenicht et al. (2012) and Chen et al. (2013) found that Akaike's information criterion (AIC; Akaike, 1974) and Bayesian information criterion (BIC; Schwarz, 1978) perform really well at selecting the correct Q-matrix among competing matrices. In this vein, AIC and BIC always selected the correct Q-matrix when a three-attribute model was estimated for data generated from a five-attribute model, and vice versa (Kunina-Habenicht et al., 2012). Regarding absolute fit indices, Chen et al. (2013) proposed to inspect the residuals between the observed and predicted proportion correct of individual items (*p*_*j*_), between the observed and predicted Fisher-transformed correlation of item pairs ($r_{jj^{\prime }}$), and between the observed and predicted log-odds ratios of item pairs ($l_{jj^{\prime }}$). Specifically, they used the *p*-value associated to the maximum *z*-scores of *p*_*j*_, $r_{jj^{\prime }}$, and $l_{jj^{\prime }}$ to evaluate absolute fit. While *p*_*j*_ obtained very bad overall results, $r_{jj^{\prime }}$ and $l_{jj^{\prime }}$ performed appropriately at identifying both Q-matrix and CDM misspecification, with a tendency to be conservative.

The aforementioned studies pointed out that these fit indices are promising for identifying the most appropriate Q-matrix. However, further research is required to examine their systematic performance in selecting the most appropriate number of attributes across a wide range of conditions. The use of fit indices to select the most appropriate model among a set of competing models, from 1 to *K* number of attributes, requires the calibration of *K* CDMs, each of them requiring a specified Q-matrix. This task demands an unfeasible amount of effort if done by domain experts, but it is viable if done by empirical means. The idea of using an empirical Q-matrix estimation method to generate Q-matrices for different number of attributes and then compare their model-fit has been already suggested by Chen et al. (2015). Furthermore, the edina package (Balamuta et al., 2020a) of the R software (R Core Team, 2020) incorporates a function to perform a Bayesian estimation of a DINA model (Chen et al., 2018) with different number of attributes, selecting the best model according to the BIC. In spite of these previous ideas, the performance of fit indices in selecting the best model among different number of attributes has not been evaluated in a systematic fashion, including both reduced (e.g., DINA) and general (e.g., G-DINA) CDMs. More details about the specific procedure used in the present study for assessing the number of attributes using model comparison are provided in the Method section.

## Goals of the Current Study

The main goal of the present study is to compare the performance of several dimensionality assessment methods in determining the generating number of attributes in CDM. Additionally, following the approach of Auerswald and Moshagen (2019), the combined performance of the methods is also evaluated to explore whether a more accurate combination rule can be obtained and recommended for applied settings. As a secondary goal, the effect of a comprehensive set of independent variables and their interactions over the accuracy of the procedures is systematically evaluated.

Table 1 provides the basis for establishing some hypotheses related to the performance of the methods. First, while CDMs are discrete latent variable models, most methods do not consider the existence of latent variables (PA with principal components extraction, MAP, EKC, EGA) or consider the existence of continuous latent variables (VSS, DETECT). The violation of this assumption might not be too detrimental, given that PA with component analysis violates EFA assumptions and is the current gold standard. On the other hand, both essential unidimensionality and simple structure assumptions are expected to have a great disruptive effect, since CDMs are usually highly multidimensional and often contain multidimensional items. Accordingly, VSS with *v* = 1 (VSS_1_) and DETECT are expected to perform poorly. Although VSS with complexity *v* > 1 is not technically assuming a simple structure (understood as a single attribute being measured by each item), its performance is still expected to be poor because of its stiffness and inability to adapt to the usual complex structure (i.e., items measuring a different number of attributes) of CDM items. Even though the remaining methods (i.e., PA, EKC, MAP, and EGA) do not assume a simple structure, their performance under complex structures remains mostly unexplored. Assessing the dimensionality of complex structures is expected to be more challenging compared to simple structures, in a similar fashion as correlated factors are more difficult to extract than orthogonal factors. The extent to which the performance of these methods is robust under complex structures is unknown. All in all, and considering the assumptions of each method, PA, EKC, MAP, and EGA, as well as the CDM model comparison approach based on fit indices (MC), are expected to perform relatively well, except for their idiosyncratic weakness conditions found in the available literature as previously described. Finally, the performance of FF is difficult to predict due to its dependency on the training samples. Even though no training samples were generated based on discrete latent variables in Goretzko and Bühner (2020), the great overall performance and generalizability of the FF model to conditions different from the ones used to train the model might extend to CDM data as well.

## Methods

### Dimensionality Estimation Methods

Eight different dimensionality estimation methods, with a total of 18 variants, were used in the present simulation study. The following text describes the specific implementation of each method.

#### Parallel Analysis

Four variants of PA were implemented as a function of the correlation matrix type (r = Pearson; ρ = tetrachoric) and the reference eigenvalue criterion (m = mean; 95 = 95th percentile): PA_rm_, PA_r95_, PA_ρ*m*_, and PA_ρ95_. All variants were implemented with principal components extraction and 100 random samples generated by random column permutation (Garrido et al., 2013; Lubbe, 2019). The sirt package (Robitzsch, 2020) was used to estimate the tetrachoric correlations for PA, as well as for the remaining methods that also make use of tetrachoric correlations.

#### Minimum Average Partial

MAP indices were based on the squared partial tetrachoric correlations computed with the psych package (Revelle, 2019). The maximum number of dimensions to extract was set to 9 (same for VSS, DETECT, and MC), so there was room for overestimating the number of attributes (the details of the simulation study are provided in the Design subsection).

#### Very Simple Structure

VSS was computed using tetrachoric correlations and the psych package. In addition to the most common VSS with complexity *v* = 1 (VSS_1_), VSS with complexity *v* = 2 (VSS_2_) was also explored.

#### Dimensionality Evaluation to Enumerate Contributing Traits

The DETECT index was computed using the sirt package, which uses the hierarchical Ward algorithm (Roussos et al., 1998) for clustering the items.

#### Empirical Kaiser Criterion

EKC was implemented by using tetrachoric correlations and the semTools package (Jorgensen et al., 2019).

#### Exploratory Graph Analysis

Two variants of EGA were implemented: EGA with GGM (EGA_G_) and EGA with TMFG (EGA_T_). The EGAnet package (Golino and Christensen, 2020) was employed for computing both variants.

#### Factor Forest

The R code published by Goretzko and Bühner (2020) at *Open Science Framework*^1^ was used for the implementation of the FF model trained in their original paper. With this code, FF can recommend between one and eight factors to retain.

#### Model Comparison Based on Fit Indices

The MC procedure was implemented varying the number of attributes from 1 to 9 as follows. First, the DFL Q-matrix estimation method (Wang et al., 2018) using Oblimin oblique rotation, tetrachoric correlations, and the row dichotomization criterion was used to specify the initial Q-matrix, and the Hull validation method (Nájera et al., 2020) using the PVAF index was then implemented to refine it and provide the final Q-matrix. Second, a CDM was fitted to the data using the final Q-matrix with the GDINA package (Ma and de la Torre, 2020b). The CDM employed to fit the data was the same as the generating CDM (i.e., DINA or G-DINA). This resulted in a set of nine competing models varying in *K*. Third, the models were alternatively compared with the AIC, BIC, and $r_{jj^{\prime }}$ fit indices. For the AIC and BIC criteria, the model with the lowest value was retained. Regarding $r_{jj^{\prime }}$, the number of items with some significant pair-wise residual (after using Bonferroni correction at the item-level) was counted. Then, the most parsimonious model with the lowest count was retained. The MC procedure with AIC, BIC, or $r_{jj^{\prime }}$ will be referred to as MC_AIC_, MC_BIC_, and MC_r_, respectively. Given that the MC procedures rely on empirically specified Q-matrices, their performance will greatly depend on the quality of such Q-matrices. Even though the DFL and Hull methods have provided good results in previous studies, their combined performance should be evaluated to examine the quality of their suggested Q-matrices. For this reason, the proportion of correctly specified q-entries was computed for the estimated (i.e., DFL) and validated (i.e., DFL and Hull) Q-matrices (more details are provided in the Dependent variables subsection). The further the DFL and Hull methods are from a perfect Q-matrix recovery, the greater the room for improvement for the MC procedures. In this vein, the set of nine competing models (using DFL and Hull) were additionally compared to the model using the generating Q-matrix, with the purpose of providing an upper-limit performance for the MC methods when the Q-matrix is perfectly recovered. The results of these comparisons will be referred to as MC_AIC−G_, MC_BIC−G_, and MC_r−G_.

### Design

Table 2 shows the factors (i.e., independent variables) used in the simulation study: number of attributes (*K*), item quality (*IQ*), sample size (*N*), ratio of number of items to attribute (*JK*), underlying correlation among the attributes (*AC*), and attribute thresholds (*AT*). The levels of each factor were selected in pursuit of representativeness of varying applied settings. For instance, the most common number of attributes (*K*) seen in applied studies is 4 (Sessoms and Henson, 2018), while 5 is the most usual value in simulation studies (e.g., de la Torre and Chiu, 2016; Ma and de la Torre, 2020a). The levels selected for item quality (*IQ*), sample size (*N*), and ratio of number of items to attribute (*JK*) are also considered as representative of applied settings (Nájera et al., 2019; Ma and de la Torre, 2020a). Regarding the attribute correlations, some applied studies have obtained very high attribute correlation coefficients, up to 0.90 (Sessoms and Henson, 2018). It can be argued that these extremely high correlations may be indeed a consequence of overestimating the number of attributes, where one attribute has been split into two or more undifferentiated attributes. For this reason, we decided to use *AC* levels similar to those used in EFA simulation studies (e.g., Garrido et al., 2013). Additionally, different attribute thresholds (*AT*) levels were included to generate different degrees of skewness in the data, given its importance in the performance of dimensionality assessment methods (Garrido et al., 2013). Finally, both a reduced CDM (i.e., DINA) and a general CDM (i.e., G-DINA) were used to generate data. A total of 972 conditions, resulting from the combination of the factor levels, were explored.

**Table 2:** Summary of the factors explored in the simulation study.

**Factors**	**Factor levels**
Number of attributes (*K*)	4, 5, 6
Item quality (*IQ*)	0.40, 0.60, 0.80
Sample size (*N*)	500, 1,000, 2,000
Ratio of number of items to attribute (*JK*)	4, 8
Correlation among the attributes (*AC*)	0, 0.30, 0.60
Attribute thresholds (*AT*)	0, 0.50, 1
Generating model (*M*)	DINA, G-DINA

### Data Generation

One hundred datasets were generated per condition. Examinees' responses were generated using either the DINA or G-DINA model. Attribute distributions were generated using a multivariate normal distribution with mean equal to 0 for all attributes. All underlying attribute correlations were set to the corresponding *AC* condition level. Attribute thresholds, which are used to dichotomize the multivariate normal distribution to determine the mastery or non-mastery of the attributes, were generated following an equidistance sequence of length *K* between –*AT* and *AT*. This results in approximately half of the attributes being “easier” (i.e., higher probabilities of attribute mastery) and the other half being “more difficult” (i.e., lower probabilities of attribute mastery). For instance, for *AT* = 0.50 and *K* = 5, the generating attributes thresholds were {−0.50, −0.25, 0, 0.25, 0.50}.

Item quality was generated by varying the highest and lowest probabilities of success, which correspond to the latent classes that master all, *P*(**1**), and none, *P*(**0**), of the attributes involved in an item, respectively. These probabilities were drawn from uniform distributions as follows: *P*(**0**)~*U*(0, 0.20) and *P*(**1**)~*U*(0.80, 1) for high-quality items, *P*(**0**)~*U*(0.10, 0.30) and *P*(**1**)~*U*(0.70, 0.90) for medium-quality items, and *P*(**0**)~*U*(0.20, 0.40) and *P*(**1**)~*U*(0.60, 0.80) for low-quality items. The expected value for the item quality across the *J* items is then 0.80, 0.60, and 0.40 for high, medium, and low-quality items, respectively. For the G-DINA model, the probabilities of success for the remaining latent classes were simulated randomly, with two constraints. First, a monotonicity constraint on the number of attributes was applied. Second, the sum of the δ parameters associated to each attribute was constrained to be higher than 0.15 to ensure the relevance of all the attributes (Nájera et al., 2020).

The Q-matrices were generated randomly with the following constraints: (a) each Q-matrix contained, at least, two identity matrices; (b) apart from the identity matrices, each attribute was measured, at least, by another item; (c) the correlation between attributes (i.e., Q-matrix columns) was lower than 0.50; (d) the proportion of one-, two-, and three-attribute items was set to 0.50, 0.40, and 0.10. Constrains (a) and (b) are in line with the identifiability recommendations made by Xu and Shang (2018). Constrain (c) ensures non overlapping attributes. Finally, constrain (d) was based on the proportion of items measuring one-, two-, and three-attributes encountered in previous literature. We examined the 36 applied studies included in the literature revision by Sessoms and Henson (2018) and extracted the complexity of the q-vectors from the 17 studies that reported the Q-matrix (see Table 3). The reason why we used a higher proportion of one-attribute items was to preserve constrain a). For instance, in the condition of *JK* = 4, at least 50% of one-attribute items are required to form two identity matrices.

**Table 3:** Complexity of q-vectors in applied studies (percentages).

	***q* = 1**	***q* = 2**	***q* = 3**	***q* > 3**	***q* = *K***
Mean	34.9	42.7	14.2	8.2	1.5
Median	30.4	43.5	10.8	0	0

*(q = 1, 2, 3) = q-vectors measuring 1, 2, or 3 attributes, respectively; (q > 3) = q-vectors measuring more than 3 attributes; (q = K) = q-vectors measuring all the attributes included in the Q-matrix*.

### Dependent Variables

Four dependent variables were used to assess the accuracy of the dimensionality assessment methods. The hit rate (*HR*) was the main dependent variable, computed as the proportion of correct estimates:

(6) $$\begin{array}{l} HR=\frac{\sum I\left(\hat{K}=K\right)}{R} \end{array}$$

where *I* is the indicator function, $\hat{K}$ is the recommended number of attributes, *K* is the generating number of attributes, and *R* is the number of replicates per condition (i.e., 100). A *HR* of 1 indicates a perfect accuracy, while an *HR* of 0 indicates complete lack of accuracy. Additionally, given that a model selection must be done according to both empirical and theoretical criteria, it is a recommended approach to examine alternative models to the one suggested by a dimensionality assessment method (e.g., Fabrigar et al., 1999). The close hit rate (*CHR*) was assessed to explore the proportion of times that a method recommended a number of attributes close to the generating number of attributes:

(7) $$\begin{array}{l} CHR=\frac{\sum I\left[\left(K-1\right)\leq \hat{K}\leq \left(K+1\right)\right]}{R} \end{array}$$

Finally, the mean error (*ME*) and root mean squared error (*RMSE*) were explored to assess the bias and inaccuracy of the methods:

(8) $$\begin{array}{l} ME=\frac{\sum \left(\hat{K}-K\right)}{R} \end{array}$$

(9) $$\begin{array}{l} RMSE=\sqrt{\frac{\sum {\left(\hat{K}-K\right)}^{2}}{R}} \end{array}$$

A *ME* of 0 indicates lack of bias, while a negative or positive *ME* indicates a tendency to underestimate or overestimate the number of attributes, respectively. It is important to note that an *ME* close to 0 can be achieved either by an accurate method, or by a compensation of under- and overestimation. On the contrary, *RMSE* can only obtain positive values: the further from 0, the greater the inaccuracy of a method.

Univariate ANOVAs were conducted to explore the effect of the factors on the performance of each method. The dependent variables for the ANOVAs were the hit rate, close hit rate, bias, and absolute error, which correspond to the numerators of Equations (6)–(9) (i.e., *HR, CHR, ME*, and *RMSE* at the replica-level), respectively. Note that the *RMSE* computed at the replica level is the absolute error (i.e., $\left|\hat{K}-K\right|$). Effects with a partial eta-squared ($\eta_{p}^{2}$) higher than 0.060 and 0.140 were considered as medium and large effects, respectively (Cohen, 1988).

In order to explore the performance of the combination rules (i.e., two or more methods taken together), the agreement rate (*AR*) was used to measure the proportion of conditions under which a combination rule recommended the same number of attributes, while the agreement hit rate (*AHR*) was used to measure the proportion of correct estimations among those conditions in which an agreement has been achieved:

(10) $$\begin{array}{l} AR=\frac{\sum I\left({\hat{K}}_{1}={\hat{K}}_{2}\right)}{R} \end{array}$$

(11) $$\begin{array}{l} AHR=\frac{\sum I\left({\hat{K}}_{1}=K|{\hat{K}}_{1}={\hat{K}}_{2}\right)}{\sum I\left({\hat{K}}_{1}={\hat{K}}_{2}\right)} \end{array}$$

where ${\hat{K}}_{1}$ and ${\hat{K}}_{2}$ are the recommended number of attributes by any two different methods. Note that these formulas can be easily generalized for more than two methods. Both a high *AR* and *AHR* are required for a combination rule to be satisfactory, since this indicates that it will be accurate and often applicable (Auerswald and Moshagen, 2019).

Finally, for the MC methods, when the model under exploration had the same number of attributes as the generating number of attributes, the Q-matrix recovery rate (*QRR*) was explored to assess the accuracy of the DFL and Hull methods. Specifically, it reflects the proportion of correctly specified q-entries. A *QRR* of 1 indicates perfect recovery. The higher the *QRR*, the closer the methods based on model-fit indices (e.g., MC_AIC_) should be to their upper-limit performance (e.g., using the generating Q-matrix as in MC_AIC−G_). All simulations and analyses were conducted using the R software. The data were simulated using the GDINA package. The codes are available upon request.

## Results

Before describing the main results, the results for the *QRR* are detailed. The overall *QRR* obtained after implementing both the DFL and Hull method was 0.949. The lowest and highest *QRR* among the factor levels were obtained with *IQ* = 0.40 (*QRR* = 0.890) and *IQ* = 0.80 (*QRR* = 0.985), respectively. These results are consistent with Nájera et al. (2020). The DFL method alone (i.e., before validating the Q-matrix with the Hull method) led to a good overall accuracy (*QRR* = 0.939). However, despite this high baseline, the Hull method led to a *QRR* improvement across all factor levels (Δ*QRR* = [0.005, 0.013]).

Table 4 shows the overall average results, across all conditions, for all the variants and dependent variables considered. The four PA variants, FF, and MC_AIC_ performed reasonably well, with a *HR* > 0.700 and a *CHR* > 0.900. EGA_G_ also obtained a high *CHR* (*CHR* = 0.918), but a much lower *HR* (*HR* = 0.576). The highest *HR* was obtained by PA_rm_ (*HR* = 0.829), while the highest *CHR* was provided by MC_AIC_ (*CHR* = 0.954). Congruently with these results, the PA variants, FF, MC_AIC_, and EGA_G_ showed a low *RMSE* (*RMSE* <1), being the MC_AIC_ the method with the lowest error (*RMSE* = 0.633). The remaining methods (i.e., MAP, VSS_1_, VSS_2_, DETECT, EKC, EGA_T_, MC_BIC_, and MC_r_) obtained a poorer performance (*HR* ≤ 0.682 and *CHR* ≤ 0.853). Regarding the bias, most methods showed a tendency to underestimate the number of attributes, especially MAP, VSS_1_, and EGA_T_ (*ME* ≤ −0.831). On the contrary, EKC and DETECT showed a tendency to overestimate the number of attributes (*ME* ≥ 1.043). Among the methods with low *RMSE*, FF (*ME* = −0.191), PA_rm_ (*ME* = −0.144), and, especially, MC_AIC_ (*ME* = 0.010), showed a very low bias. Finally, the MC methods that rely on the generating Q-matrix (i.e., MC_AIC−G_, MC_BIC−G_, MC_r−G_) generally provided good results, outperforming their corresponding MC method. Specifically, MC_AIC−G_ obtained the highest overall accuracy (*HR* = 0.886; *CHR* = 0.975; *RMSE* = 0.458).

**Table 4:** Overall performance for all dimensionality estimation methods.

**Method**	***HR***	***CHR***	***ME***	***RMSE***
PA_rm_	0.829	0.947	−0.144	0.681
PA_r95_	0.801	0.919	−0.309	0.876
PA_ρ*m*_	0.805	0.938	−0.190	0.734
PA_ρ95_	0.770	0.904	−0.369	0.941
MAP	0.518	0.618	−1.337	2.205
VSS_1_	0.278	0.378	−1.045	3.135
VSS_2_	0.424	0.522	−0.040	2.339
DETECT	0.492	0.691	1.043	1.956
EKC	0.502	0.625	2.200	3.988
EGA_G_	0.576	0.918	−0.257	0.870
EGA_T_	0.337	0.782	−0.831	1.231
FF	0.824	0.918	−0.191	0.746
MC_AIC_	0.768	0.954	0.010	**0.633**
MC_BIC_	0.682	0.824	−0.620	1.289
MC_r_	0.635	0.853	0.096	1.027
———————————————————————————————————				
MC_AIC−G_	0.886	0.975	−0.022	**0.458**
MC_BIC−G_	0.713	0.829	−0.593	1.270
MC_r−G_	0.814	0.922	−0.086	0.774

The dashed line separates the MC methods that are implemented using the generating Q-matrix (i.e., MC_AIC−G_, MC_BIC−G_, MC_r−G_). Best results for HR, CHR, and RMSE are shown in bold, considering the MC methods with the generating Q-matrix separately. HR ≥ 0.700 and CHR ≥ 0.900 results are underlined. HR, hit rate; CHR, close hit rate; ME, mean error; RMSE, root mean squared error; PA_r_, parallel analysis with Pearson correlations; PA_ρ_, parallel analysis with tetrachoric correlations; PA_m_, parallel analysis with mean eigenvalue criterion; PA_95_, parallel analysis with 95th percentile eigenvalue criterion; MAP, minimum average partial; VSS_1_, very simple structure with complexity v = 1; VSS_2_, very simple structure with complexity v = 2; DETECT, dimensionality evaluation to enumerate contributing traits; EKC, empirical Kaiser criterion; EGA_G_, exploratory graph analysis with Gaussian graphical model; EGA_T_, exploratory graph analysis with triangulated maximally filtered graph; FF, factor forest; MC_AIC_, model comparison based on AIC; MC_BIC_, model comparison based on BIC; MC_r_, model comparison based on the Fisher-transformed correlations; MC_*−G_, model comparison using the generating Q-matrix when the number of attributes coincides with the generating number of attributes.

Table 5 shows the results for the methods that obtained the best overall performance as indicated by *CHR* > 0.900 (i.e., PA_rm_, EGA_G_, FF, and MC_AIC_) across the factor levels. Only PA_rm_ is shown among the PA variants because their results were congruent and PA_rm_ obtained the better overall performance. In addition to these methods, the MC_AIC−G_ is also included to provide a comparison with MC_AIC_. The results from Table 5 can be easier interpreted by inspecting the main effect size values obtained in the ANOVAs (see Table 6). These main effects offer a proper summary of the results since only one interaction effect, which will be described below, was relevant ($\eta_{p}^{2}$ > 0.140).

**Table 5:** Performance of the best methods by factor level.

	***K***			***IQ***			***N***			***JK***		***AC***			***AT***			***M***	
**Method**	**4**	**5**	**6**	**0.40**	**0.60**	**0.80**	**500**	**1,000**	**2,000**	**4**	**8**	**0**	**0.30**	**0.60**	**0**	**0.50**	**1**	**D**	**G-D**
***Hit rate (HR)***																			
PA_rm_	**0.870**	**0.828**	0.789	**0.606**	**0.911**	0.970	0.726	**0.844**	**0.918**	**0.772**	**0.886**	0.895	0.905	0.687	0.857	0.838	**0.793**	**0.810**	0.848
EGA_G_	0.662	0.571	0.494	0.447	0.625	0.655	0.543	0.590	0.594	0.525	0.627	0.607	0.591	0.529	0.565	0.586	0.576	0.443	0.709
FF	0.858	0.785	**0.827**	0.596	0.894	**0.981**	**0.780**	0.823	0.868	0.768	0.879	0.897	0.832	**0.741**	**0.883**	**0.843**	0.745	0.803	0.845
MC_AIC_	0.721	0.788	0.794	0.552	0.835	0.916	0.719	0.774	0.811	0.754	0.782	0.812	0.774	0.718	0.811	0.772	0.721	0.791	0.745
MC_AIC−G_	**0.899**	**0.893**	**0.866**	**0.675**	**0.984**	**0.999**	**0.796**	**0.895**	**0.967**	**0.863**	**0.909**	0.895	0.890	**0.872**	**0.907**	**0.897**	**0.853**	**0.966**	0.806
***Close hit rate (CHR)***																			
PA_m_	**0.973**	0.949	0.921	0.860	**0.983**	**0.999**	0.900	0.959	**0.983**	0.913	0.982	0.979	0.983	0.879	0.942	0.951	**0.949**	0.947	0.948
EGA_G_	0.951	0.929	0.875	0.855	0.954	0.946	0.898	0.926	0.931	0.902	0.934	0.944	0.934	0.877	0.879	0.934	0.942	0.891	0.945
FF	0.936	0.961	0.858	0.787	0.969	**0.999**	0.886	0.918	0.950	0.854	0.983	0.960	0.926	0.869	0.939	0.924	0.891	0.916	0.921
MC_AIC_	0.949	**0.970**	**0.942**	**0.891**	0.980	0.989	**0.928**	**0.961**	0.972	**0.948**	0.960	0.966	0.957	**0.938**	**0.964**	**0.956**	0.941	**0.948**	**0.959**
MC_AIC−G_	**0.979**	**0.981**	**0.963**	**0.924**	**0.999**	**1**	**0.945**	**0.982**	**0.997**	**0.968**	0.981	0.978	0.977	**0.969**	**0.980**	**0.979**	**0.965**	**0.982**	**0.967**
***Mean error (ME)***																			
PA_rm_	−0.076	−0.136	−0.219	−0.325	−0.100	−0.006	−0.228	−0.134	−0.068	−0.271	−0.016	0.100	−0.044	−0.487	−0.174	−0.148	−0.109	−0.150	−0.137
EGA_G_	−0.007	−0.266	−0.498	−0.412	−0.275	−0.083	−0.240	−0.249	−0.281	−0.507	−0.007	−0.164	−0.217	−0.389	−0.169	−0.291	−0.310	−0.282	−0.232
FF	−0.128	−0.258	−0.188	−0.430	−0.137	−0.008	−0.161	−0.227	−0.186	−0.309	−0.074	−0.126	−0.202	−0.245	−0.104	−0.178	−0.292	−0.217	−0.166
MC_AIC_	0.232	0.006	−0.207	−0.203	0.140	0.095	−0.107	0.021	0.118	−0.124	0.145	0.038	0.031	−0.037	0.047	0.042	−0.057	−0.129	0.150
MC_AIC−G_	0.067	−0.003	−0.129	−0.069	0.004	0.001	−0.069	0.002	0.003	−0.127	0.084	0.011	−0.012	−0.064	0.007	−0.009	−0.063	−0.056	0.013
***Root mean squared error (RMSE)***																			
PA_rm_	**0.491**	0.661	0.844	1.098	**0.389**	0.184	0.926	0.619	**0.386**	0.858	**0.436**	**0.447**	0.403	1.014	0.708	0.656	**0.677**	0.690	0.672
EGA_G_	0.749	0.839	1.003	1.076	0.737	0.754	0.940	0.840	0.826	0.928	0.808	0.777	0.817	0.999	0.978	0.817	0.804	1.002	0.713
FF	0.685	0.663	0.871	1.201	0.452	**0.149**	0.884	0.724	0.604	0.921	0.514	0.520	0.693	0.958	0.635	0.708	0.875	0.759	0.733
MC_AIC_	0.671	**0.555**	**0.668**	**0.925**	0.477	0.348	**0.748**	**0.600**	0.533	**0.669**	0.596	0.555	0.616	**0.719**	**0.562**	**0.624**	0.706	**0.642**	**0.625**
MC_AIC−G_	**0.416**	**0.405**	**0.539**	**0.781**	**0.134**	**0.024**	**0.646**	**0.411**	**0.205**	**0.513**	**0.395**	**0.425**	0.441	**0.502**	**0.406**	**0.429**	**0.528**	**0.339**	**0.551**

*Best results for HR, CHR, and RMSE are shown in bold, considering MC_AIC−G_ separately. Results for HR, CHR, and RMSE that are higher than MC_AIC−G_ are also underlined. K, number of attributes; IQ, item quality; N, sample size; JK, ratio of the number of items to attribute; AC, correlation among the attributes; AT, attribute thresholds; M, generating model; D, DINA model; G-D, G-DINA model; PA_rm_, parallel analysis with Pearson correlations and mean eigenvalue criterion; EGA_G_, exploratory graph analysis with Gaussian graphical model; FF, factor forest; MC_AIC_, model comparison based on AIC; MC_AIC−G_, model comparison based on AIC and using the generating Q-matrix when the number of attributes coincides with the generating number of attributes*.

**Table 6:** Univariate ANOVAs main effect size values ($\eta_{p}^{2}$).

	***K***	***IQ***	***N***	***JK***	***AC***	***AT***	***M***
***Hit rate***							
PA_rm_	0.014	**0.251**	0.076	0.041	0.117	0.009	0.005
EGA_G_	0.024	0.042	0.003	0.014	0.006	0.000	0.085
FF	0.009	**0.218**	0.013	0.031	0.041	0.034	0.005
MC_AIC_	0.009	**0.161**	0.011	0.001	0.012	0.011	0.004
MC_AIC−G_	0.005	**0.344**	0.103	0.012	0.002	0.013	0.131
***Close hit rate***							
PA_rm_	0.014	0.109	0.037	0.036	0.068	0.000	0.000
EGA_G_	0.016	0.031	0.003	0.004	0.014	0.012	0.011
FF	0.040	**0.161**	0.015	0.084	0.030	0.009	0.000
MC_AIC_	0.004	0.051	0.010	0.001	0.004	0.003	0.001
MC_AIC−G_	0.003	0.059	0.023	0.002	0.001	0.002	0.003
***Bias***							
PA_rm_	0.015	0.073	0.019	0.067	**0.216**	0.003	0.000
EGA_G_	0.074	0.035	0.001	0.111	0.018	0.008	0.001
FF	0.007	0.069	0.002	0.032	0.006	0.014	0.002
MC_AIC_	0.126	0.094	0.037	0.075	0.005	0.010	0.080
MC_AIC−G_	0.048	0.009	0.009	0.078	0.007	0.007	0.009
***Absolute error***							
PA_rm_	0.026	**0.260**	0.089	0.064	**0.145**	0.002	0.002
EGA_G_	0.029	0.056	0.005	0.013	0.014	0.005	0.071
FF	0.008	**0.223**	0.017	0.054	0.045	0.024	0.002
MC_AIC_	0.006	**0.167**	0.017	0.002	0.013	0.011	0.001
MC_AIC−G_	0.007	**0.280**	0.090	0.011	0.003	0.010	0.074

*$\eta_{p}^{2}$ > 0.060 and $\eta_{p}^{2}$ > 0.140 are shown underlined and bolded, respectively. K, number of attributes; IQ, item quality; N, sample size; JK, ratio of the number of items to attribute; AC, correlation among the attributes; AT, attribute thresholds; M, generating model; D, DINA model; G-D, G-DINA model; PA_rm_, parallel analysis with Pearson correlations and mean eigenvalue criterion; EGA_G_, exploratory graph analysis with Gaussian graphical model; FF, factor forest; MC_AIC_, model comparison based on AIC; MC_AIC−G_, model comparison based on AIC and using the generating Q-matrix when the number of attributes coincides with the generating number of attributes*.

Regarding the hit rate, *IQ* was the factor that most affected all the methods ($\eta_{p}^{2}$ ≥ 0.161), except for EGA_G_. These methods performed very accurately with *IQ* = 0.80 (*HR* ≥ 0.916), but poorly with *IQ* = 0.40 (*HR* ≤ 0.675). Other factors obtained a medium effect size for one specific method. EGA_G_ was affected by *M*, obtaining a higher accuracy with the G-DINA model (*HR*= 0.709) than with the DINA model (*HR* = 0.443). On the other hand, PA_rm_ was affected by *N* and *AC*. PA_rm_ obtained the highest *HR* in most conditions, especially with large sample sizes (*N* = 2000), but was negatively affected by high correlations among the attributes (*AC* = 0.60). In the cases in which PA_rm_ was not the best performing method, the FF obtained the highest accuracy. FF obtained the biggest advantage in comparison to the other methods under *N* = 500 (Δ*HR* = 0.054). Results for the *RMSE* showed a very similar pattern to those from *HR*. The most notable difference was that MC_AIC_ obtained the lowest *RMSE* under most conditions, especially *AC* = 0.60 (|Δ*RMSE*| = 0.239). On the contrary, PA_rm_ showed a smaller error with lower attribute correlations, especially *AC* = 0.30 (|Δ*RMSE*| = 0.213).

The close hit rate of the methods was more robust than the *HR* to the different simulation conditions. Only PA_rm_ and, especially, FF were affected by *IQ*. PA_rm_ was also affected by *AC*, and FF by *JK*. The *CHR* of both EGA_G_ and MC_AIC_ remained stable across the factor levels. The highest *CHR* was obtained by MC_AIC_ in most conditions, obtaining the biggest advantage under *AC* = 0.60 (Δ*CHR* = 0.059). PA_rm_ and FF provided the highest *CHR* in those conditions in which MC_AIC_ did not obtain the best result.

With respect to the bias (i.e., *ME*), Table 6 shows that the only large effect was observed for *AC* on PA_rm_. However, there was a relevant effect for the interaction between *AC* and *IQ* ($\eta_{p}^{2}$ = 0.194). This was the only interaction with a large effect among all the ANOVAs. Namely, the strong tendency to underestimate seen for PA_rm_ under *AC* = 0.60 was mainly due to *IQ* = 0.40. Thus, under *IQ* = 0.40, PA_rm_ showed a strong tendency to underestimate when *AC* = 0.60 (*ME* = −1.108), but a slight tendency to overestimate when *AC* = 0 (*ME* = 0.258). With *IQ* ≥ 0.60 and *AC* ≤ 0.30, PA_rm_ obtained a low bias (*ME* ≤ |0.055|). Apart from this interaction, other factors with relevant effect sizes were: a) *K*, which had an effect on EGA_G_ and MC_AIC_; b) *IQ*, with an effect on PA_rm_, FF, and MC_AIC_; and c) *JK*, which had an effect on PA_rm_, EGA_G_, and MC_AIC_. In general, the most demanding levels of these factors (i.e., *K* = 6, *IQ* = 0.40, *JK* = 4) led to an underestimation tendency for the methods. Finally, while PA_rm_, EGA_G_, and FF showed a negative bias (i.e., *ME* <0) across almost all conditions, MC_AIC_ showed a positive bias (i.e., *ME* > 0) under several conditions, especially *K* = 4 and the G-DINA model (*ME* ≥ 0.150).

Finally, MC_AIC−G_ performed the best under almost all conditions and dependent variables, with the only exception of *AC* ≤ 0.30 and G-DINA generated data, where PA_rm_ obtained slightly better results. As expected, MC_AIC−G_ outperformed MC_AIC_ under all conditions. The ANOVA effects were similar for both methods. One of the main differences is that the *HR* of MC_AIC−G_ was more affected by the sample size (a steeper *HR* improvement as *N* increased) and the generating model (performing comparatively better under the DINA model). On the other hand, the *ME* of MC_AIC−G_ was more robust under different levels of *K* and *M*.

Table 7 shows the results for the combination rules split by sample size. VSS_1_, VSS_2_, DETECT, EKC, and EGA_T_ are not included because they were not usually consistent with any other method (i.e., *AR* <0.50). Both the *AR* and the *AHR* tended to increase as the sample size increased. As expected from the results above, the best performing combination rules were mainly formed by PA (especially PA_rm_), FF, and MC (especially MC_AIC_). The combination rule formed by PA_rm_ and FF obtained arguably the best balance between agreement and accuracy (*AR* ≥ 0.70; *AHR* ≥ 0.923), while FF and MC_AIC_ obtained a higher accuracy with a slightly lower agreement (*AR* ≥ 0.65; *AHR* ≥ 0.953). The best accuracy was obtained by the combination rule formed by MC_AIC_ and MAP (*AHR* ≥ 0.980), although at the cost of a lower agreement (*AH* ≈ 0.46). In addition to these two-method combination rules, the performance of the three best methods (i.e., PA_rm_, FF, and MC_AIC_) taken together was also explored. This combination rule showed a very high overall accuracy while keeping an *AR* > 0.50. Specifically, for *N* = 500, 1000, and 2000, it obtained *AHR (AR)* = 0.976 (0.57), 0.985 (0.65), and 0.992 (0.70), respectively.

**Table 7:** Performance of the combination rules by sample size.

	**PA_***rm***_**	**PA_***r*95**_**	**PA_***ρm***_**	**PA_**ρ95**_**	**MAP**	**EGA_***G***_**	**FF**	**MC_***AIC***_**	**MC_***BIC***_**	**MC_***r***_**
***N*** **=** **500**										
PA_r95_	**0.816** (0.80)									
PA_ρ*m*_	**0.732** (0.93)	**0.789** (0.80)								
PA_ρ95_	**0.828** (0.74)	**0.697** (0.92)	**0.777** (0.79)							
MAP	0.939 (0.52)	0.876 (0.55)	0.926 (0.50)	0.854 (0.53)						
EGA_G_	0.848 (0.54)	0.841 (0.52)	0.837 (0.51)	*0.833* (0.49)	0.904 (0.38)					
FF	**0.923** (0.70)	**0.896** (0.70)	0.913 (0.67)	0.885 (0.67)	0.935 (0.53)	0.903 (0.52)				
MC_AIC_	0.914 (0.66)	0.912 (0.64)	0.911 (0.62)	0.910 (0.60)	*0.980* (0.47)	*0.862* (0.49)	0.953 (0.65)			
MC_BIC_	0.885 (0.61)	0.843 (0.62)	0.873 (0.58)	0.834 (0.59)	0.849 (0.57)	*0.837* (0.45)	0.873 (0.62)	0.884 (0.59)		
MC_r_	0.893 (0.56)	0.861 (0.56)	0.887 (0.53)	0.854 (0.53)	*0.915* (0.42)	*0.847* (0.40)	0.915 (0.56)	0.849 (0.61)	0.843 (0.52)	
Single	0.726	0.687	0.687	0.646	0.507	0.543	0.780	0.719	0.572	0.584
***N*** **=** **1,000**										
PA_r95_	**0.891** (0.89)									
PA_ρ*m*_	**0.849** (0.96)	**0.877** (0.89)								
PA_ρ95_	**0.897** (0.84)	**0.821** (0.95)	**0.866** (0.88)							
MAP	0.974 (0.53)	0.953 (0.54)	0.974 (0.51)	0.946 (0.52)						
EGA_G_	0.900 (0.61)	0.885 (0.60)	0.884 (0.60)	0.871 (0.59)	*0.899* (0.41)					
FF	**0.957** (0.79)	0.929 (0.80)	**0.948** (0.77)	**0.916** (0.78)	0.960 (0.54)	0.911 (0.58)				
MC_AIC_	**0.951** (0.73)	**0.947** (0.72)	**0.950** (0.71)	0.946 (0.69)	*0.994* (0.46)	0.910 (0.52)	**0.967** (0.70)			
MC_BIC_	**0.933** (0.70)	**0.904** (0.72)	0.927 (0.69)	0.899 (0.69)	0.934 (0.55)	0.866 (0.52)	**0.900** (0.73)	0.930 (0.65)		
MC_r_	0.936 (0.63)	0.926 (0.62)	0.934 (0.61)	0.924 (0.59)	*0.984* (0.38)	*0.879* (0.44)	0.945 (0.60)	0.861 (0.67)	0.908 (0.56)	
Single	0.844	0.815	0.818	0.781	0.522	0.590	0.823	0.774	0.684	0.645
***N*** **=** **2,000**										
PA_r95_	**0.937** (0.95)									
PA_ρ*m*_	**0.925** (0.98)	**0.935** (0.95)								
PA_ρ95_	**0.944** (0.92)	**0.907** (0.97)	**0.929** (0.95)							
MAP	0.985 (0.53)	0.980 (0.53)	0.990 (0.52)	0.982 (0.52)						
EGA_G_	0.935 (0.62)	0.922 (0.62)	0.927 (0.62)	0.911 (0.62)	*0.883* (0.41)					
FF	**0.972** (0.86)	**0.956** (0.87)	**0.969** (0.86)	**0.950** (0.87)	0.974 (0.54)	0.911 (0.61)				
MC_AIC_	**0.978** (0.78)	**0.977** (0.77)	**0.978** (0.77)	**0.978** (0.75)	*0.998* (0.45)	0.953 (0.50)	**0.983** (0.73)			
MC_BIC_	**0.966** (0.80)	**0.950** (0.81)	**0.964** (0.79)	**0.949** (0.79)	0.985 (0.53)	0.890 (0.57)	**0.928** (0.81)	**0.960** (0.70)		
MC_r_	0.970 (0.65)	0.968 (0.64)	0.970 (0.65)	0.968 (0.63)	*0.996* (0.36)	*0.932* (0.41)	0.974 (0.62)	**0.866** (0.72)	0.952 (0.59)	
Single	0.918	0.900	0.909	0.884	0.526	0.594	0.868	0.811	0.791	0.675

*Each cell shows the agreement hit rate (AHR) and the agreement rate (AR; within the parentheses) for each combination rule. The AHR of combination rules with AR > 0.70 are shown in bold, and those with AR <0.50 are shown in italics. PA_r_, parallel analysis with Pearson correlations; PA_ρ_, parallel analysis with tetrachoric correlations; PA_m_, parallel analysis with mean eigenvalue criterion; PA_95_, parallel analysis with 95th percentile eigenvalue criterion; MAP, minimum average partial; EGA_G_, exploratory graph analysis with Gaussian graphical model; FF, factor forest; MC_AIC_, model comparison based on AIC; MC_BIC_, model comparison based on BIC; MC_r_, model comparison based on the Fisher-transformed correlations; N, sample size*.

## Real Data Example

**Figure 2:**
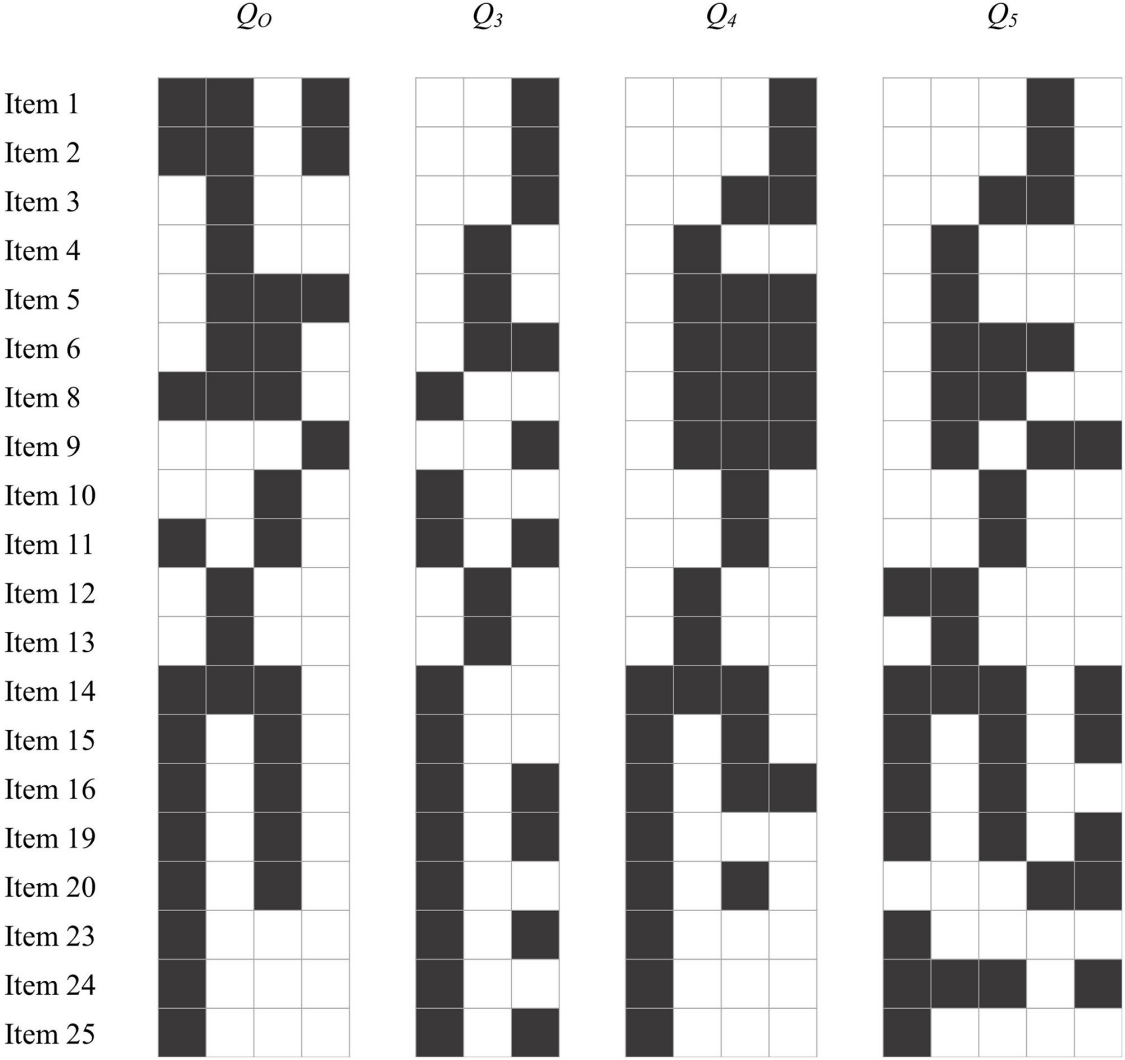
Q-matrices of the real data example. White cells represent *q*_*jk*_ = 0 and black cells represent *q*_*jk*_ = 1. *Q*_*O*_ is the Q-matrix from Chen et al. (2020); *Q*_3_ to *Q*_5_ are the suggested Q-matrices by DFL and Hull methods from 3 to 5 attributes.

Real data were analyzed to illustrate the performance of the dimensionality estimation methods explored in the simulation study. This section can be also understood as an illustration of how to approach the problem of determining the number of attributes in applied settings. The data employed for this example was previously analyzed by Chen et al. (2020). The dataset consists of dichotomous responses from 400 participants to 20 items from an intelligence test. Each item consists of nine matrices forming a 3 rows × 3 columns disposition, in which the ninth matrix (i.e., the lower right) is missing. Participants must select the missing matrix out of eight possible options. There are no missing data. The dataset is available at the edmdata package (Balamuta et al., 2020b) and item definitions can be found at *Open Psychometrics*.^2^ Chen et al. (2020) defined four attributes involved in the test: (a) learn the pattern from the first two rows and apply it to the third row, (b) infer the best overall pattern from the whole set of matrices, (c) recognize that the missing matrix is different from the given matrices (e.g., applying rotations or stretching), and (d) recognize that the missing matrix is exactly as one of the given matrices. The authors did not explicitly define a Q-matrix for this dataset because they focused on the exploratory estimation of the item parameters. However, they described a procedure to derive a Q-matrix from the item parameter estimates by dichotomizing the standardized coefficients related to each attribute (Chen et al., 2020, pp. 136). This original Q-matrix, which is here referred to as *Q*_*O*_, is shown in Figure 2.

According to the findings from the simulation study, the following steps are recommended to empirically determine the number of attributes in CDM data: (a) if PA_rm_, FF, and MC_AIC_ agree on their suggestion, retain their recommended number of attributes; (b) if any two of these methods agree, retain their recommended number of attributes; (c) if none of these methods agree, explore the recommended number of attributes by those that suggest a similar (i.e., ±1) number of attributes; (d) if these methods strongly disagree, explore the recommended number of attributes by each of them. Constructing several Q-matrices is a very challenging and time-consuming process for domain experts; thus, the Q-matrices suggested by the DFL and Hull methods (which are already used to implement the MC methods), can be used as a first approximation. Domain experts should be consulted to contrast the interpretability of these Q-matrices.

All the dimensionality assessment methods included in the simulation study were used to assess the number of attributes of the dataset. Their recommendations were as follows: 1 attribute was retained by MAP and VSS_1_; 2 attributes by PA_ρ95_, PA_ρ*m*_, VSS_2_, and EGA_T_; 3 attributes by PA_rm_, PA_r95_, and MC_BIC_; 4 attributes by EGA_G_, MC_AIC_, and MC_r_; 5 attributes by EKC and FF; and 8 attributes by DETECT. In accordance with the simulation study results, MAP, VSS_1_, and EGA_T_, which showed a tendency to underestimate, suggested a low number of attributes, while DETECT, which showed a strong tendency to overestimate, suggested the highest number of attributes.

Following the previously described guidelines, we focused on the recommendations of PA_rm_, MC_AIC_, and FF (i.e., 3, 4, and 5 attributes, respectively). Step *c* of the guidelines apply to this case because none of the methods agreed on their suggestion, but PA_rm_ and MC_AIC_, as well as MC_AIC_ and FF, recommended a close number of attributes. Thus, we explored solutions from 3 to 5 attributes in terms of model fit. A G-DINA model was fitted using each of the Q-matrices from 3 to 5 attributes (*Q*_3_*-Q*_5_) suggested by the DFL and Hull methods (see Figure 2). Additionally, *Q*_*O*_ was also used to fit a G-DINA model for comparison purposes. Table 8 shows the fit indices for each model. Overall, *Q*_4_ obtained the best model fit. This result is in agreement with the number of attributes defined by Chen et al. (2020). Thus, a solution with four attributes was considered the most appropriate. The differences between *Q*_4_ and *Q*_*O*_ were not very pronounced: 81.25% of the q-entries were the same for both matrices. In an applied study in which no original Q-matrix had been prespecified, *Q*_4_ could be used as a starting point for domain experts to achieve a Q-matrix specification that provides both good fit and theoretical interpretability.

**Table 8:** Model-fit for the real data illustration Q-matrices.

	**−2LL**	***np***	**AIC**	**BIC**	**min.p(*r*)**	**items(*r*)**
*Q_3_*	8,584	59	8,702	**8,938**	0.006	5
*Q_*O*_*	8,498	97	8,692	9,079	0.000	8
*Q_4_*	8,441	97	**8,635**	9,022	**0.098**	**0**
*Q_5_*	8,390	133	8,656	9,187	**0.096**	**0**

*Best result for AIC, BIC, and items(r), as well as min.p(r) > 0.05, are shown in bold. −2LL, deviance; np, number of parameters; AIC, Akaike's information criterion; BIC, Bayesian information criterion; min.p(r), minimum p-value (adjusted for multiple comparisons) associated to the residual Fisher-transformed correlations; items(r), number of items showing a statistically significant (adjusted for multiple comparisons) Fisher-transformed correlation with at least another item. Q_3_, Q_4_, Q_5_, Q-matrix specified by DFL and Hull methods with 3, 4, and 5 attributes, respectively; Q_O_, Q-matrix from Chen et al. (2020)*.

## Discussion

The correct specification of the Q-matrix is a prerequisite for CDMs to provide accurate attribute profile classifications (Rupp and Templin, 2008; Gao et al., 2017). Because the Q-matrix construction process is usually conducted by domain experts, many Q-matrix validation methods have been recently developed with the purpose of empirically evaluating the decisions made by the experts. Additionally, empirical methods to specify the Q-matrix directly from the data (i.e., Q-matrix estimation methods), without requiring a previously specified one, have been also proposed. The problem with the Q-matrix estimation and validation methods proposed so far is that they do not question the number of attributes specified by the researcher. The assumption of known dimensionality has not been exhaustively explored in the CDM framework. This contrasts with the vast literature on dimensionality assessment methods in the factor analysis framework, where this problem is considered of major importance and has received a high degree of attention (e.g., Garrido et al., 2013; Preacher et al., 2013). All in all, the main goal of the present study was to explore the performance of several dimensionality assessment methods from the available literature in determining the number of attributes in CDMs. A comprehensive simulation study was conducted with that purpose.

Results from the simulation study showed that some methods available can be considered suitable for assessing the dimensionality of CDMs. Namely, parallel analysis with principal components and random column permutation (i.e., PA), the machine learning factor forest model (i.e., FF), and using the AIC fit index to compare CDMs with different number of attributes (i.e., MC_AIC_) obtained high overall accuracies (*HR* ≥ 0.768). PA with Pearson correlations and mean eigenvalue criterion (i.e., PA_rm_) obtained the highest overall accuracy, while MC_AIC_ obtained the best close accuracy, considering a range of ±1 attribute around the generating number of attributes. Item quality was found to be the most relevant simulation factor, severely affecting the performance of PA_rm_, FF, and MC_AIC_. Thus, the percentage of correct estimates varied from around 60% with low-quality items to more than 90% with high-quality items. Apart from item quality, PA_rm_ was also affected by the sample size and the correlation among the attributes, showing a bad performance with highly correlated attributes. These results are in line with previous studies (e.g., Garrido et al., 2013; Lubbe, 2019). MC_AIC_ and, especially, FF, were more robust to the different explored conditions (other than item quality). However, it should be noted that, unlike PA_rm_ and FF (which consistently tended to underestimate the number of attributes under almost all conditions), MC_AIC_ bias might show a slightly under- or overestimation tendency depending on the number of attributes, item quality, ratio of number of items to attribute, and generating model.

The remaining methods (i.e., MAP, VSS_1_, VSS_2_, DETECT, EKC, EGA_G_, EGA_T_, MC_BIC_, and MC_r_) obtained an overall poor performance, and thus their use cannot be recommended for the assessment of CDM data dimensionality. Of these methods, DETECT and EKC showed a heavy tendency to overestimate. Even though EKC was expected to perform better, it was observed that the first reference eigenvalue was usually very high, leaving the remaining ones at low levels. These resulted in the EKC often performing identically to what the Kaiser-Guttman criterion would (which is known for its tendency to overestimate the number of dimensions). On the other hand, MAP, VSS_1_, EGA_T_, and MC_BIC_ showed a strong tendency to underestimate. Even though a higher performance was expected for MAP and EGA_T_, their underestimation tendency is aligned with previous findings (Garrido et al., 2011; Golino et al., 2020). As for the MC methods, while both AIC and BIC have shown good results in selecting the correct Q-matrix among competing misspecified Q-matrices (Kunina-Habenicht et al., 2012; Chen et al., 2013), it is clear that the higher penalization that BIC applies compared to AIC is not appropriate for the dimensionality assessment problem. Finally, EGA_G_ was the only remaining method that obtained a good performance in terms of close hit rate. However, its overall hit rate was low, especially due to its poor performance when the generating model was the DINA model.

Although the influence of the generating model was most noticeable for EGA_G_, most dimensionality assessment methods from the EFA framework performed worse under the DINA model than under the G-DINA model. These results might be due to the non-compensatory nature of the DINA model, in which the relationship between the number of mastered attributes and the probability of correctly answering an item clearly deviates from being linear (in a more pronounced way that under the G-DINA model, as illustrated in Figure 1). A greater depart from linearity might produce a greater disruption to the performance of all the methods that are based on correlations (e.g., PA, FF, EGA). On the contrary, the MC methods performed better under the DINA model. Since the MC methods are precisely modeling the response process, they benefit from the parsimony of reduced models. The performance of the dimensionality assessment methods under other commonly used reduced CDMs (e.g., the *deterministic inputs, noisy “or” gate* model or DINO; Templin and Henson, 2006) is expected to follow a similar pattern as the one obtained for the DINA model.

An important finding regarding the MC methods is that the performance of the variants that made use the generating Q-matrix (e.g., *HR* = 0.886 for MC_AIC−G_) was notably better than that of their corresponding methods (e.g., *HR* = 0.768 for MC_AIC_). Given that the Q-matrices specified by the DFL and Hull methods obtained a very high overall recovery rate (*QRR* = 0.949), these results imply that a small improvement in the quality of the Q-matrices might have a big impact on the dimensionality assessment performance of the MC procedures. This reiterates the importance of applying empirical Q-matrix validation methods such as the Hull method, even though the improvement over the original Q-matrix (be it empirically estimated or constructed by domain experts) might seem small.

The exploration of combination rules showed that PA_rm_ and FF often agreed on the recommended number of attributes (*AR* ≥ 0.70), providing a very high combined accuracy (*AHR* ≥ 0.923). FF and MC_AIC_ obtained an even higher accuracy (*AHR* ≥ 0.953) with a slightly lower agreement rate (*AR* ≥ 0.65). When these three methods agree on their number of attributes, which occurred in more than 60% of the overall conditions, the percentage of correct estimations was, at least, of 97.6%. Given these results, the following guidelines can be followed when aiming to empirically determine the number of attributes in CDM data: (a) if PA_rm_, FF, and MC_AIC_ agree on their suggestion, retain their recommended number of attributes; (b) if any two of these methods agree, retain their recommended number of attributes; (c) if none of these methods agree, explore the recommended number of attributes by those that suggest a similar (i.e., ±1) number of attributes; (d) if these methods strongly disagree, explore the recommended number of attributes by each of them. The number of attributes provided by the dimensionality assessment methods should be understood as suggestions; the final decision should consider theoretical interpretability as well.

These guidelines were used to illustrate the dimensionality assessment procedure using a real dataset. The number of suggested number of attributes greatly varied from 1 attribute (MAP and VSS_1_) to 8 attributes (DETECT). The best three methods from the simulation study, PA_rm_, MC_AIC_, and FF recommended 3, 4, and 5 attributes, respectively. After inspecting the model fit of the Q-matrices suggested by the DFL and Hull methods from 3 to 5 attributes, it was found that 4 was the most appropriate number of attributes, which was consistent with Chen et al. (2020). The interpretability of the Q-matrices suggested by the DFL and Hull method should be further explored by domain experts, who should make the final decision on the Q-matrix specification.

The present study is not without limitations. First, the CDMs used to generate the data (i.e., DINA and G-DINA) were also used to estimate the models in the MC methods. In applied settings, the saturated G-DINA model should be used for both estimating/validating the Q-matrix and assessing the number of attributes to make sure that there are no model specification errors. After these two steps have been fulfilled, item-level model comparison indices should be applied to check whether more reduced CDMs are suitable for the items (Sorrel et al., 2017). The main reason why the DINA model was used to estimate the models in the MC methods (whenever the generating model was also the DINA model) was to try to reduce the already high computation time of the simulation study. Nevertheless, it is expected that the results of these conditions would have been similar if the G-DINA model were used to estimate these models: it provides similar results as the DINA model given that the sample size is not very small (i.e., *N* <100; Chiu et al., 2018). Second, the generalization of the results to other conditions not considered in the present simulation study should be done with caution. For instance, the range of the number of attributes was kept around the most common number of attributes encountered in applied settings and simulation studies. Highly dimensional scenarios (e.g., *K* = 8) were not explored because the computation time increases exponentially with the number of attributes and the simulation study was already computationally expensive. Hence, the performance of the dimensionality assessment methods under highly dimensional data should be further evaluated. In this vein, an important discussion might arise when considering highly dimensional CDM data. As Sessoms and Henson (2018) reported, many studies obtained attribute correlations higher than 0.90. These extremely high correlations imply that those attributes are hardly distinguishable, which might indicate that the actual number of attributes underlying the data is lower than what has been specified. It can be argued that CDM attributes are expected to show stronger correlations than EFA factors because attributes are usually defined as fine-grained skills or concepts within a broader construct. However, it is important to note that each attribute should be still distinguishable from the others. Otherwise, the interpretation of the results might be compromised. The proper identification of the number of attributes might be of help in this matter.

Finally, only one of the best three performing methods (i.e., FF) can be directly implemented by the interested researcher in assessing the dimensionality of CDM data, using publicly available functions. With the purpose of facilitating the application of the other two best performing methods, the specific implementations of parallel analysis and model comparison approach used in the present study have been included in the cdmTools R package (Nájera et al., 2021). A sample R code to illustrate a dimensionality assessment study of CDM data can be found in Supplementary Materials.

## Data Availability Statement

Publicly available datasets were analyzed in this study. This data can be found at: https://cran.r-project.org/web/packages/edmdata.

## Author Contributions

PN wrote the R simulation scripts, performed the real data analyses, and wrote the first draft of the manuscript. FA and MS wrote sections of the manuscript. All authors contributed to conception, design of the study, manuscript revision, read, and approved the submitted version.

## Conflict of Interest

The authors declare that the research was conducted in the absence of any commercial or financial relationships that could be construed as a potential conflict of interest.

## Footnotes

^1^https://osf.io/mvrau/

^2^https://openpsychometrics.org/_rawdata/
