# Supplementary material for: Determining the Number of Attributes in Cognitive Diagnosis Modeling
Source: Front Psychol. 2021 Feb 15;12:614470. doi: 10.3389/fpsyg.2021.614470 (PMC7917061; doi:10.3389/fpsyg.2021.614470)
Supplement: Supplementary file 1 [file Data_Sheet_1.DOCX]

Supplementary Material

# Dimensionality assessment illustration for CDM data with R software

To implement the factor forest (FF) model, please download the *Factor_Forest.zip* folder from <https://osf.io/rs28q/> (Goretzko & Bühner, 2020).

To install the cdmTools R package (Nájera, Sorrel, & Abad, 2021), use the following commands:

# install.packages("devtools")

devtools::install_github("Pablo-Najera/cdmTools")

Updates and more information about the cdmTools package can be found at <https://github.com/Pablo-Najera/cdmTools>.

The following example code uses simulated data from the GDINA R package (Ma & de la Torre, 2020) to illustrate the implementation of the FF method, the parallel analysis with Pearson correlations and mean eigenvalue criterion (PA_rm_), and the model comparison approach using the AIC (MC_AIC_).

#-------------------

# 1. Simulated data

#-------------------

library(GDINA)

Q <- sim30GDINA$simQ # generating K = 5

dat <- sim30GDINA$simdat

#-------------------

# 2. Factor forest

#-------------------

# 2.1. Load the required functions and objects (set the proper working directory to read the files located at the Factor_Forest.zip folder)

source("xgb-functions.R")

source("CD_approach.R")

source("factor.forest.R")

xgb <- readRDS(file = "tunedxgb.rds")

# 2.2. Load the required packages

library(mlr)

library(psych)

library(ineq)

library(BBmisc)

library(ddpcr)

# 2.3. Apply the factor forest method

factor.forest(newdata = dat, mod = xgb) # suggested K = 5

#----------------------

# 3. Parallel analysis

#----------------------

# 3.1. Load the required packages

library(cdmTools)

# 3.2. Apply the parallel analysis

res.paK <- paK(dat, cor = "cor")

res.paK$sug.K # suggested K = 5

res.paK$plot

#----------------------

# 4. Model comparison

#----------------------

# 4.1. Load the required packages

# library(cdmTools)

# 4.3. Apply the model comparison method

res.mcK <- modelcompK(dat, rangeK = 1:7)

res.mcK$sug.K["AIC"] # suggested K = 5

**References**

Goretzko, D., & Bühner, M. (2020). One model to rule them all? Using machine learning algorithms to determine the number of factors in exploratory factor analysis. *Psychological Methods*. DOI: 10.1037/met0000262.

Ma, W., & de la Torre, J. (2020). GDINA: An R package for cognitive diagnosis modeling. *Journal of Statistical Software*, *93*, 1–26. DOI: 10.18637/jss.v093.i14

Nájera, P., Sorrel, M. A., & Abad, F. J. (2021). *cdmTools: Useful Tools for Cognitive Diagnosis Modeling. R package version 0.1.1*. Retrieved from https://github.com/Pablo-Najera/cdmTools.
